# The impact of age on long QT syndrome

**DOI:** 10.18632/aging.102623

**Published:** 2019-12-28

**Authors:** Norbert Guettler, Kim Rajappan, Edward Nicol

**Affiliations:** 1 Section Head, Internal Medicine and Cardiology, Air Force Centre of Aerospace Medicine, Fuerstenfeldbruck, Germany; 2 Consultant Cardiologist and Electrophysiologist, Central Military Hospital, Koblenz, Germany; 3 Consultant Cardiologist and Electrophysiologist, Cardiac Department, John Radcliffe Hospital, Oxford University Hospitals NHS Foundation Trust, Oxford, UK; 4 Consultant Cardiologist, Department of Cardiology, Royal Brompton Hospital, London, UK

**Keywords:** inherited long QT syndrome (LQTS), childhood, adolescence, aging, gender

Inherited long QT syndrome (LQTS) is an arrhythmogenic disorder predisposing to sudden cardiac death (SCD) secondary to polymorphic VT; mostly torsades de pointes. The mean age at presentation is 14 years of age, whilst the median age of individuals who die of LQTS is 32 years, with men predominantly affected. Inherited LQTS is characterized by prolonged ventricular repolarization represented on the ECG by an increased corrected QT (QTc) interval. Three main genes account for approximately 90% of all genotyped cases of LQTS. The risk of arrhythmic events is determined by age, sex, QTc interval, a history of syncope, and genotype, and in acquired LQTS QTc prolongation secondary to certain medication further increases the risk of cardiac arrhythmias (http://crediblemeds.org).

Children and adolescents constitute an important risk group for inherited LQTS. During childhood, the risk of cardiac events is significantly higher in LQT1 males than in LQT1 females (hazard ratio [HR] = 1.72), whereas there is no significant gender-related difference among LQT2 and LQT3 carriers [1]. During adulthood, LQT1 females (HR = 3.35) and LQT2 females (HR = 3.71) have a significantly higher risk of cardiac events than age-matched males. The lethality of cardiac events is highest in LQT3 males and females (19% and 18%), and higher in LQT1 and LQT2 males (5% and 6%) than in LQT1 and LQT2 females (2% for both).

The analysis of serial QTc interval measurements in a large cohort of children and adolescents with LQT1 and LQT2 demonstrated two main findings. Firstly, both LQT1 and LQT2 male patients show significant QTc interval shortening after the onset of puberty; in LQT2 male patients, this is preceded by a progressive QTc interval prolongation. Secondly, the age of 12 to 14 years is an important transitional period in which differences between males and females for both genotypes are seen, ages broadly corresponding with the onset of puberty [2]. The authors concluded that for risk stratification and management, clinicians should be aware of these significant age-, sex-, and genotype-related trends in QTc interval, especially the important role of the onset of puberty. LQT2 patients seem to be more sensitive to variations of sex hormone concentrations than LQT1 patients.

A recent analysis of the QTc interval in healthy individuals during childhood and adolescence [3] observed a gradual QTc shortening in males (by about 15 ms) during the ages of 13 – 19 years coinciding with the development of secondary sex signs. This is in contrast to a gradual QTc prolongation in females (by about 10 ms) during ages 16 – 19 years which is not related to the development of secondary sex signs. It appears that QTc changes in males during puberty are probably caused by hormonal shifts, whilst the QTc changes seen in females might have a more complex aetiology.

In older patients, aging is associated with increased prevalences of atrial and ventricular arrhythmias, reflecting disruption of the normal sequence of ion channel activation and inactivation generating the propagated cardiac action potential. In addition to these biochemical and electrophysiological changes, aging is associated with structural changes, particularly myocardial fibrosis. This is relevant for LQTS3 patients, who account for 5 to 10% of cases of congenital LQTS, whose LQT phenomenon is caused by a gain-of-function mutation in the SCN5A gene, which encodes the α subunit of the Na^+^ channel (Nav1.5). LQTS3 patients show increased risk of life-threatening ventricular arrhythmias, particularly after 40 years of age, consistent with ion channel abnormalities caused by aging [4,5].

The QTc interval in general increases with age, with the age-related increase is more evident in men than in women. This suggests that male sex does not afford protection against potentially fatal arrhythmias at older age and there is a need for an increased awareness of the QTc prior to the use of QTc prolonging drugs, e. g. psychotropics, and the need to evaluate the QTc after initiation of therapy [6]. Additionally, it has been shown that coronary heart disease augments the risk for LQTS-related cardiac events [7].

In conclusion both inherited and acquired LQTS are influenced by age. The onset of puberty is an important transitional period for inherited LQTS, which affects QTc interval and clinical risk, probably in part secondary to sex hormone shifts. Individuals with inherited LQTS remain at higher risk for life-threatening cardiac events after age 40 years. Risk factors for SCD include age-specific factors, gender, clinical history, and the LQTS genotype. QTc generally increases with age, and age-related electrophysiological and structural changes may increase the risk of potentially life-threatening arrhythmic events. Consequently, clinicians treating individuals above age 40, with inherited or acquired QT prolongation need an increased awareness of their QTc prior to, and during, therapy with drugs that have the potential to prolong the QTc interval.
